# Mental health and adaptive functioning among school-aged children living with HIV in Zambia

**DOI:** 10.3389/fpsyt.2022.922944

**Published:** 2022-09-07

**Authors:** Lisa Kalungwana, Susan Malcolm-Smith, Leigh Schrieff

**Affiliations:** ^1^Department of Psychology, University of Zambia, Lusaka, Zambia; ^2^Applied Cognitive Science and Experimental Neuropsychology Team, Department of Psychology, University of Cape Town, Cape Town, South Africa

**Keywords:** mental health, HIV, adaptive function, children, low- to middle-income countries

## Abstract

**Background:**

The number of children living with HIV (CLWHIV) has been increasing, reflected by lower mortality. However, this change is coupled with higher rates of morbidity, where CLWHIV face considerable challenges, including neurocognitive delays and mental health and behavioral functioning challenges. Despite Sub-Sahara accounting for the highest number of CLWHIV, there is still limited research on the effects of HIV on child mental health and adaptive functioning.

**Method:**

Mental health and adaptive functioning were assessed in 120 children. The sample included 62 CLWHIV and 58 demographically-matched HIV-uninfected children aged 6–12 years. Mental health was assessed using the Connors, while adaptive functioning was assessed using the Vineland Adaptive Behavioral Scale (VABS).

**Results:**

Scores obtained were within average ranges for mental health (T-score 40–59) and adaptive functioning standard scores (70–115). However, CLWHIV had significantly higher mental health problems than uninfected children in executive functioning and aggressiveness (*p* < 0.05). CLWHIV had lower adaptive functioning scores on the VABS Communication domain although these differences were not significant. In the Daily Living Skills domain, CLWHIV had significantly higher scores than the HIV-uninfected children (*p* < 0.05). There were no significant differences in the Socialization subdomain. Furthermore, CLWHIV had significantly higher scores on the Maladaptive Behavior scales of the VABS' internalizing and externalizing subdomains.

**Conclusion:**

Challenges to mental health and adaptive functioning are still pervasive among CLWHIV. These findings support the need to develop support mechanisms for CLWHIV to help address mental health and adaptive functioning problems, especially as they progress into adolescence.

## Background

It is estimated that about 22.5 million people live with HIV in Sub-Saharan Africa (SSA) (1). Approximately 1.8 million children under the age of 15 live with HIV, globally, and 80% of these children live in SSA (2). It is estimated that there are currently 72 000 Zambian children living with HIV (3). It was previously reported that 49 000 children living with HIV (CLWHIV) in Zambia do not have access to ART (4). Although access to ART has undoubtedly reduced mortality, children diagnosed with HIV tend to suffer developmental delays; they are also prone to mental health problems and poor adaptive functioning (5).

Poor mental health and adaptive functioning have been highlighted among CLWHIV, particularly in high-income countries [HICs; (6)]. In low- to middle-income countries (LMICs), the incidence of mental health problems has also been documented; however, most of the studies among CLWHIV did not include a comparative HIV-uninfected sample to ascertain whether mental health problems are comparable (7). Despite not including comparative samples, studies on mental health among CLWHIV in LMICs have shown that there is a high incidence of mental health problems that are associated with several challenges, including poor access to health services, lack of youth-friendly mental health services, and the inability to continually access treatment as they transition to adult hospitals, which all affect treatment adherence in the long run (8, 9). Comparing mental health problems among CLWHIV with HIV-uninfected children offers better understanding of mental health among younger children. Studies in HICs that have evaluated mental health problems among CLWHIV have shown lower rates of mental health problems than community prevalence. The lower rates of mental health problems in CLWHIV are attributed to the fact that CLWHIV have regular clinical visits and tend to benefit more from early detection and management of mental health problems than HIV-uninfected children in the community. Therefore, a better understanding of mental health problems among CLWHIV and uninfected children can inform treatment options that might be used to manage mental health problems, particularly in low-income environments where access to mental health services is limited. Furthermore, mental health studies in CLWHIV have mostly been conducted among older children and adolescents, with limited research on mental health problems among younger children (10).

The effects of pediatric HIV go beyond mental health and may affect other areas of a child's well-being. Adaptive functioning is an area of functioning that has been identified as a key factor that is relevant to identifying the competencies and abilities of children diagnosed with developmental and intellectual disabilities (11). Adaptive functioning generally relates to areas such interpersonal communication and activities of daily living that include a broad spectrum of activities including personal hygiene and care. It also includes competencies in community living, academic achievement skills, and effectively managing the necessities of a personal ecological setting (12, 13).

Studies on adaptive functioning among CLWHIV in HICs have had mixed findings, with some reporting no differences in adaptive functioning between CLWHIV and uninfected children (14), while other studies have indicated poorer adaptive functioning scores in CLWHIV (15). Nonetheless, studies on adaptive functioning among CLWHIV particularly in HICs have shown that poor adaptive functioning puts children at risk of poorer outcomes in other areas of life including academic achievement and neurocognitive functioning (14–16). In LMICs studies have largely been conducted among HIV-affected households rather than CLWHIV specifically, and these results tend to reflect poorer behavioral functioning, poorer health outcomes and challenges in cognitive functioning (17, 18). An adaptive functioning study in South Africa established that, similar to studies in HICs, CLWHIV with poor adaptive functioning have poorer neurocognitive functioning and poor academic achievement (19).

With the growing number of CLWHIV surviving into adolescence and adulthood, it is important to consider the challenges in mental health and adaptive functioning that these children may encounter. This study thus aimed to establish whether HIV affects the mental health and adaptive functioning of school-aged children living with HIV compared to HIV-uninfected children in Zambia.

## Materials and methods

The study took place in Lusaka, the capital city of Zambia. The data was collected between September, 2015 and December, 2016. Two peri-urban clinics were included as study sites. The clinics selected are centers that provide services to CLWHIV and Prevention of Mother to Child Transmission (PMTCT) services. The target population included children of primary school age (6–12 years). The study employed a cross-sectional quasi- experimental design that compared two pre-existing groups, CLWHIV and an HIV-uninfected matched control group. CLWHIV were recruited from ART centers at the participating clinics. Community health workers identified the HIV-uninfected children from the clinic in the outpatient department. They approached mothers attending PMTCT clinics and the pediatric outpatient department at the participating clinics and enquired whether they have children who might want to participate in the study. All children identified as exposed to but uninfected by HIV were not included in the study. There was no known relationship between the CLWHIV and the HIV-uninfected children. Routine HIV testing at every clinic visit is mandatory in Zambia; therefore, an HIV-negative test was not taken from the uninfected children, as it was based on the standard testing conducted at the participating medical facility. The CLWHIV were unaware of their HIV status as medical guidelines do not recommend the disclosure of HIV status to children below the age of 12 who were the target age group (20).

For both the CLWHIV and the HIV-uninfected controls, exclusion criteria included previous neurological conditions such as epilepsy, cerebral Malaria, cerebral palsy, infantile meningitis, and a previous head injury that had led to a loss of consciousness. Other exclusion criteria for the CLWHIV-group and HIV-uninfected group included acute illness or hospital admission at cognitive assessment and inability to perform neuropsychological tests.

Written informed consent was obtained from parents/caregivers of children participating in the study, and children and their parents/caregivers were informed that participation in the study was voluntary. Ethical approval was received from the ERES Converge IRB in Zambia. Additional consent was obtained from the Ministry of Community Development, Mother and Child Health, and the Lusaka District Health Medical Office to access clinics where recruitment was carried out.

Data was collected by two trained psychology graduates. The research assistants went through an additional training by a neuropsychologist on the administration of the measures used in the assessment as well as on working with CLWHIV and their caregivers. The data collected was further evaluated by two senior neuropsychologists to ensure that it was done correctly and based on standardized procedures of administration and scoring. The data collection process was carried out in English as this is the official language of the country.

### Mental health measures

We used the Conners 3, a revised version of the Conners Rating Scales (21) to assess mental health and behavioral functioning. It is a well-standardized and widely used assessment tool to diagnose Attention-Deficit/Hyperactivity Disorder (ADHD) and other developmental disorders in children and adolescents. The test has three versions available in both a full version and a short form: Parent and Teacher Rating Scales which can be used for children aged 6 to 18 years, and a Self-reporting scale for children aged 8 to 18 years. In this research, the Parent Rating Short form was used, containing 45 items in which parents/caregivers rate their children's behavior on a five-point Likert scale from *not at all* to *most of the time*. There are six subtests: Inattention, Hyperactivity/Impulsivity, Learning Problems, Executive Functioning, Defiance/Aggression, and Peer relations. The information provided triangulates the child's behavior at home, at school, in social settings, and interactions with peers (21). Although not previously used in Zambia, the test has been successfully used in other African studies, particularly in South Africa (22, 23).

### Adaptive functioning measures

We used the Vineland Adaptive Behavior Scale (VABS) (24) to assess adaptive functioning and maladaptive, internalizing, and externalizing behavior. The scale provides subscales on children's adaptive functioning in various areas of daily living. In this version, parents/caregivers are asked to rate whether the child has performed an activity based on the following scale: “usually,” “sometimes or partially,” or “never.” The scale has been widely used in research in Zambia to assess adaptive competencies in both clinical and non-clinical samples (25–27). The subscales cover the domains of daily living skills (including items on socially appropriate feeding, toileting, and grooming), community living skills (including rule-following, reliability, respect for privacy, and understanding of rights), and social skills (including sensitivity to others' needs, prosocial attitudes, cooperation, sharing, respect for property, and good sportsmanship). The parents' interview form version of the VABS was used in this study.

### Data analysis

Data were analyzed using IBM SPSS Statistics Version 28. Independent *t*-tests were used to determine whether there were any differences in performance between CLWHIV and HIV-uninfected children. Bootstrapping was run on all between-group comparisons. Boot strapping provides reliable estimates given the small sample size and non-normality in the data through the generation of a thousand possible data sets (28). Cohen's *d* was used to establish the effect size estimate (ESE) on the observed differences between the two groups.

## Results

### Sample characteristics

The sample included 120 children aged 6–12 (*M: 9.8; SD: 1.8*) years, with 58 (45.7%) children living with HIV (CLWHIV group) and 62 matched controls (HIV-uninfected group; 54.3%; see Table 1). Fifty-six (56%) participants within the total sample were male. All the CLWHIV were vertically infected and the majority were taking ART at the study time. The majority of the participants were from a low socioeconomic status background, with most of the parents having <7 years of education and 77.5% of the families earning less than $1 per day. There were no significant differences in any of the demographic data presented in Table 1.

**Table 1:** Participant characteristics.

		**HIV status**		**Test statistics**		
		**CLWHIV**	**Uninfected**	**χ^2^/*t***	***p***	***ESE***
Sex (Male: Female)		28:30	26:36	0.693	0.490	0.501
Age (Mean: SD)		9.81:1.82	9.87:1.89	−0.179	0.858	−0.033
Parents education	No education	2 (3.5%)	1 (1.6%)			
	Grade 1–6	18 (31.6%)	13 (21%)	a	0.609	0.197
	Grade 7	17 (29.8%)	23 (37.1%)			
	Grade 8–9	9 (15.8%)	6 (9.7%)			
	Grade 10–11	4 (7%)	7 (11.3%)			
	Grade 12	5 (8.8%)	9 (14.5%)			
	13+	2 (3.5%)	3 (4.8%)			
Level of income^1^	0–1,999	41 (71.9%)	51 (82.3%)	a	0.210	0.196
	2,000–3,999	14 (24.6%)	10 (16.1%)			
	4,000–6,999	0 (0%)	1 (1.6%)			
	7,000–9,500	1 (1.8%)	0 (0%)			
	9,600–12,500	1 (1.8%)	0 (0%)			
Parents employment	Higher executive	1 (1.8%)	1 (1.6%)	a	0.221	0.210
	Business	1 (1.8%)	0 (0%)			
	Managers					
	Administrative	2 (3.5%)	1 (1.6%)			
	Clerical	0 (0%)	2 (3.2%)			
	Skilled manual	7 (12.3%)	11 (17.7%)			
	Semi-skilled	15 (26.3%)	9 (14.5%)			
	Unskilled	30 (52.6%)	33 (53.2%)			
	Homemaker	0	4 (6.5%)			
	No occupation	1 (1.8%)	1 (1.6%)			

1 Amounts presented in Zambian Kwacha. At the time of data collection, the Zambian Kwacha was trading at US$1 to ZMW 8.6.

^a^Fisher's exact test was used because >20% of cells had an expected count of <5.

### Mental health and behavioral functioning

Table 2 shows that parents/caregivers of CLWHIV reported significantly more behavioral problems for this group than parents/caregivers of the HIV-uninfected children in the Executive Functioning and Aggressiveness domains. There were no significant differences in mental health and behavioral domains of Inattention, Hyperactivity, Learning Problems or Peer Relations.

**Table 2:** Between group comparisons on Connors^a^.

**HIV status**	**Infected**			**Uninfected**			**Test statistics**		
	***M (SD)***	**Range**	***N***	**M (SD)**	**Range**	***n***	***t***	***p***	***ESE^b^***
Inattention	40.83 (1.41)	40–65	40	41.53 (2.57)	40–54	30	−0.1.363	0.185	−0.356
Hyperactivity	42.53 (2.11)	40–79	40	42.77 (1.81)	40–60	30	−0.868	0.194	−0.210
Learning problems	49.23 (6.55)	40–81	40	48.03 (6.52)	40–90	30	0.755	0.459	0.182
Executive functioning	47.85 (5.45)	40–70	40	44.53 (4.70)	40–67	30	2.669	0.006	0.645
Aggressiveness	47 (4.76)	43–90	40	44.87 (1.07)	44–77	30	2.740	0.027	0.580
Peer relations	47.00 (4.47)	44–77	40	45.83 (4.76)	44–71	30	1.051	0.320	0.254

a Higher scores in each domain are indicative of mental health challenges.

b ESE is Cohen's d (0.2-small, 0.5- medium, 0.8-large).

### Adaptive functioning

Scores of adaptive functioning on all four domains are shown in Tables 3–**6**. Table 3 shows that parents/caregivers of CLWHIV reported significantly poorer scores in the receptive language domain for this group, as compared to scores reported by parents/caregivers of the HIV-uninfected group.

**Table 3:** Between group differences on the Vineland Adaptive Behavior Scale: Communication domain^a^.

**HIV status**	**CLWHIV**			**Uninfected**			**Test statistics**		
	***M (SD)***	**Range**	***N***	**M (SD)**	**Range**	***n***	***t***	***p***	***ESE^b^***
Vineland communication	68.17 (6.06)	56–86	57	69.71 (4.88)	59–124	59	−1.24	0.200	−0.276
Vineland receptive	10.94 (1.77)	7–15	57	11.74 (1.48)	8–34	59	−2.18	0.034	−0.485
Vineland expressive	9.38 (1.54)	6–13	57	9.49 (1.46)	5–12	61	−0.333	0.757	−0.073
Vineland written	7.42 (1.86)	3–12	58	7.51 (1.33)	5–11	53	−0.278	0.786	−0.059

a Higher scores in each domain are indicative of better functioning.

b ESE is Cohen's d (0.2-small, 0.5- medium, 0.8-large).

Table 4 shows statistically significant differences in the overall Daily Living Skills subdomain between the CLWHIV group and the HIV-uninfected children. In this domain, parents/caregivers of CLWHIV reported better daily living skills for this group than the parents/caregivers of the HIV-uninfected group.

**Table 4:** Between group differences on the Vineland Adaptive Behavioral Scale: Daily living skills^a^.

**HIV status**	**CLWHIV**			**Uninfected**			**Test statistics**		
	***M (SD)***	**Range**	***N***	**M (SD)**	**Range**	***n***	***t***	***p***	***ESE^b^***
Vineland daily living skills	71.60 (8.68)	54–95	55	68.37 (6.07)	51–87	59	2.04	0.036	0.455
Vineland personal care	11.58 (2.58)	6–16	53	10.89 (1.60)	8–61	58	1.64	0.122	0.347
Vineland domestic care	10.44 (3.04)	4–17	58	9.20 (2.47)	2–17	61	1.97	0.054	0.439
Vineland community functioning	8.15 (1.14)	5–11	54	8.23 (1.89)	4–12	60	−0.229	0.821	−0.055

a Higher scores in each domain are indicative of better functioning.

b ESE is Cohen's d (0.2-small, 0.5- medium, and 0.8-large).

A similar pattern was observed in the level of coping skills on the VABS, where parents/caregivers of the CLWHIV group reported higher levels that parents/caregivers of the HIV- uninfected group (see Table 5).

**Table 5:** Between group comparisons on the Vineland Adaptive Behavioral Scale: Socialization subscale^a^.

**HIV status**	**CLWHIV**			**Uninfected**			**Test statistics**		
	***M (SD)***	**Range**	***N***	**M (SD)**	**Range**	***n***	***t***	***p***	***ESE^b^***
Vineland socialization	62.27 (5.29)	51–75	57	61.43 (5.73)	51–143	60	0.691	0.503	0.154
Vineland interpersonal relationships	7.92 (1.08)	4–10	58	8.31 (1.27)	5–13	62	−1.52	0.131	−0.339
Vineland play and leisure	7.02 (1.15)	4–10	58	7.11 (1.53)	3–11	62	−0.317	0.752	−0.070
Vineland coping skills	8.85 (1.95)	3–14	57	7.94(1.47)	5–14	62	2.316	0.022	0.515

a Higher scores in each domain are indicative of better functioning.

b ESE is Cohen's d (0.2-small, 0.5- medium, and 0.8-large).

Finally, Table 6 shows significant differences on the Maladaptive scale of the VABS in the Maladaptive, Internalization, and Externalization domains. According to the caregiver reports of the CLWHIV group, there were higher reports of maladaptive, internalizing, and externalizing behavior for this group than for the HIV-uninfected children.

**Table 6:** Between group comparisons on the Vinelands Adaptive Behavioral Functioning: Maladaptive behavior subscale^a^.

**HIV status**	**CLWHIV**			**Uninfected**			**Test statistics**		
	***M (SD)***	**Range**	***N***	**M (SD)**	**Range**	***n***	***t***	***p***	***ESE^b^***
Vinelands maladaptive behavior	12.31 (1.85)	1–16	58	12.03 (0.16)	12–13	54	3.79	0.003	0.729
Vinelands internalizing behavior	15.67 (2.41)	10–12	58	14.00 (1.53)	12–18	59	3.75	0.004	0.733
Vinelands externalizing behavior	15.21 (2.13)	8–12	57	13.60 (1.94)	12–18	62	2.98	0.003	0.634

a Higher scores in each domain are indicative of more problem behavior.

b ESE is Cohen's d (0.2-small, 0.5- medium, and 0.8-large).

## Discussion

This study was a small, cross sectional study that aimed to establish whether CLWHIV experienced more mental health and adaptive functioning problems than HIV-uninfected children. The reports of adaptive functioning and mental health and behavioral functioning were based on caregiver reports. This study shows that HIV has a significant role in the mental health, behavioral functioning, and adaptive functioning of CLWHIV when compared to demographically matched HIV-uninfected children. However, due to the limited sample size in this study, the results should be interpreted cautiously.

### Mental health and behavioral functioning

The current study evaluates mental health problems among younger CLWHIV matched demographically with a group of HIV-uninfected children. The results showed that CLWHIV had higher mental health scores (suggesting more difficulties) than the HIV-uninfected in all domains. However, these differences in scores were only significant in the executive functioning and aggressiveness domains. The results of this study are consistent with previous studies carried out in Zambia, where parents/caregivers of CLWHIV reported more mental health problems relative to established test norms, despite the norms being based on a United Kingdom population (29, 30).

Unlike most studies that looked at mental health among adolescents with HIV (ALWHIV), our study focused on younger school-aged children. A study conducted in Malawi within a similar population group showed that CLWHIV had higher caregiver reports of emotional and behavioral problems than established test norms; however, like most studies, there was no inclusion of a comparison group (10). A similar study was conducted with a much younger study group of CLWHIV between the ages of 6 and 8 in South Africa, which showed a high prevalence of mental health problems, particularly among the younger children (31). Our study equally showed higher caregiver reports of poor mental health and behavioral function among CLWHIV in aggression and executive functioning. In this study, we identified particular domains of mental health that may be of concern among younger CLWHIV in SSA. Most recommendations to date have been to implement policies in helping adolescent children transition to adult clinics and accessing mental health services. However, earlier interventions would be helpful in assisting pre-adolescent children to navigate living with HIV and to manage knowledge of disclosure and treatment adherence.

The finding that executive functioning is one of the domains affected in CLWHIV is similar to what was observed in a multisite study of caregiver depression and cognitive functioning where an association was observed between higher levels of depression in caregivers and poor executive functioning in children (32). Based on caregiver reports, CLWHIV had more executive function problems than the uninfected children. These findings align with other studies that indicate that executive function continues to be a domain of concern among young CLWHIV (33, 34).

Other studies have pointed to mental health problems being associated with low SES neighborhoods, which would predispose both CLWHIV and HIV-uninfected children to more mental health problems due to exposure to several environmental traumas (35). In this study, based on comparative scores, the parents/caregivers of CLWHIV reported higher mental health problems than the parents/caregivers of uninfected children. However, based on cut-off points set by the Conners, the parents/caregiver reports in both the study and control group had scores within the average range; therefore, the role of environment increasing mental health problems outside of HIV infection was not supported within the parameters of the current study. However, it could be argued that the Conners being interpreted against international norms may not be the most appropriate route to understanding mental health as expressed in the local Zambian community. The majority of standardized tests used in SSA are normed on western populations. The implications of the use of tests that do not have local norms are usually that the test may fail to give a clear representation of mental health to the local population, which may be argued to have been the case in this study.

### Adaptive functioning

Regarding adaptive functioning, parents/caregivers of the CLWHIV reported more difficulties in the Communication domain than parents/caregivers of the HIV-uninfected children. This result is similar to what has been reported in the literature on adaptive functioning among CLWHIV in HICs (14, 36). The communication domain on the VABS assesses how well an individual can exchange information with others. This ability extends to how well a person can process information, verbal skills, and reading and writing (37).

Our findings are similar to those observed in other studies among CLWHIV where communication skills were lower than for HIV-uninfected children or established test norms (15, 38). In our study, the receptive language domain largely accounted for this difference, and this too is similar to what was obtained in a study carried out in Canada with an immigrant sample where poor language skills were attributed to the transition to use of a second language (15). However, this was not the case in our study, as all participants used the same language and came from a similar SES background. Therefore, communication differences in the current study's groups could be attributed to the effect HIV may have on language development among children. In younger, HIV-exposed but uninfected children, poor receptive scores on the VABS have been associated with poor cognitive functioning and poor developmental outcomes (39, 40). In older ALWHIV, poor receptive skills have been associated with poor treatment adherence, primarily related to poor communication with caregivers (41). The findings in our study thus show the prevalence of poor communication skills in younger CLWHIV. Interventions in this age group would help alleviate CLWHIV's communication skills challenges as they transition through adolescence and later adulthood.

Significant differences between CLWHIV and the HIV-uninfected children were also seen in the Daily Living Skills domain, with parents/caregivers of CLWHIV reporting better performances in this domain than parents/caregivers of the HIV-uninfected children. This finding is consistent with some studies showing that CLWHIV tend to learn better home management and daily living skills as they usually take up the role of caregiver for an unwell parent or when a parent is not present (42). In this study, none of the children came from a child-headed household as they were all accompanied by a parents/caregiver; however, CLWHIV may still be taking the role of caregiver when the parents/caregivers are unwell. This finding is important as it speaks to the different needs and challenges that CLWHIV may face compared to adults and the need to create child-specific interventions relevant to specific age groups and populations.

In the maladaptive domain of the VABS, parents/caregivers of the CLWHIV group reported higher maladaptive scores than parents/caregivers of the HIV-uninfected children. Significantly higher scores were equally observed in the internalizing and externalizing subdomains than in the HIV-uninfected children. Previous findings on internalizing and externalizing behaviors among CLWHIV have been mixed. Some studies have shown that CLWHIV or their parents/caregivers report more externalizing and internalizing behavioral problems than their uninfected peers (14). However, studies in LMICs have demonstrated no significant differences in externalizing and internalizing scores between ALWHIV and established test norms (30, 43). Inconsistent results may be related to differences in measures used and methodical differences employed in the various studies. Further, most of these studies were carried out with adolescents, so differences in age groups could also account for the variations in the findings. An indication of poor or increased maladaptive behavior can point to the role that HIV may play in the developing brain among younger CLWHIV. Furthermore, early identification of problem behavior in CLWHIV would help develop suitable interventions as research has shown that in the absence of interventions, externalizing and internalizing behaviors may persist into adolescence and adulthood (44).

## Limitations

Our study was not without limitations. First, the study had a small sample size which reduced the statistical power of the data analysis, and may make generalization of the findings difficult. Second, was the fact that the study was cross-sectional and thus could not look at whether mental health problems experienced in younger children would persist into adolescence. A third limitation was the reliance on caregiver reports of mental health, behavioral, and adaptive functioning. Previous research has indicated that caregivers of CLWHIV tend to experience some mental health challenges themselves and may in turn report similar outcomes in their children (42, 45). Previous research has highlighted that parents/caregiver mental health impacts the mental health of CLWHIV (42); however, this study did not collect data to ascertain caregiver characteristics. Further studies to consider long-term outcomes of mental health status among CLWHIV in LMICs are needed.

## Conclusion

Mental health problems and poor adaptive functioning were present among CLWHIV in Zambia. Interventions for children concerning mental health need to begin early. Most interventions are designed for adolescents (46); however, this study indicates that younger children are equally vulnerable to mental health and adaptive functioning problems. Our study was one of the first studies to evaluate adaptive functioning among CLWHIV, particularly highlighting that in some cases, CLWHIV may have poor adaptive functioning, but these problems do not extend to daily living skills. Interventions in terms of adaptive functioning should therefore be targeted specifically at affected domains. Lastly, CLWHIV would benefit from early mental health screening to help learn better- coping strategies as they progress into adolescence.

## Data availability statement

The raw data supporting the conclusions of this article will be made available by the authors, without undue reservation.

## Ethics statement

The studies involving human participants were reviewed and approved by ERES Ethics Board of Zambia. Written informed consent to participate in this study was provided by the participants' legal guardian/next of kin.

## Author contributions

All authors made significant contribution in the conception, development and finalization of this manuscript.

## Conflict of interest

The authors declare that the research was conducted in the absence of any commercial or financial relationships that could be construed as a potential conflict of interest.

## Publisher's note

All claims expressed in this article are solely those of the authors and do not necessarily represent those of their affiliated organizations, or those of the publisher, the editors and the reviewers. Any product that may be evaluated in this article, or claim that may be made by its manufacturer, is not guaranteed or endorsed by the publisher.
